# Ipilimumab and nivolumab in advanced hepatocellular carcinoma after failure of prior immune checkpoint inhibitor-based combination therapies: a multicenter retrospective study

**DOI:** 10.1007/s00432-022-04206-8

**Published:** 2022-07-21

**Authors:** Daniel Roessler, Osman Öcal, Alexander B. Philipp, Daniel Markwardt, Stefan Munker, Julia Mayerle, Leonie S. Jochheim, Katharina Hammer, Christian M. Lange, Andreas Geier, Max Seidensticker, Florian P. Reiter, Enrico N. De Toni, Najib Ben Khaled

**Affiliations:** 1 Department of Medicine II, University Hospital, LMU Munich, Marchioninistrasse 15, 81377 Munich, Bavaria Germany; 2 Department of Radiology, University Hospital, LMU Munich, Munich, Germany; 3 Department for Gastroenterology and Hepatology, University Hospital Essen, University Duisburg-Essen, Duisburg, Germany; 4 Division of Hepatology, Department of Medicine II, University Hospital Würzburg, Würzburg, Germany; 5 German Cancer Consortium (DKTK), Partner site Munich, Munich, Germany; 6 Bavarian Cancer Research Center (BZKF), Partner site Munich, Munich, Germany

**Keywords:** Atezolizumab, Bevacizumab, Immune checkpoint inhibitors, Hepatocellular carcinoma, Ipilimumab, Nivolumab

## Abstract

**Introduction:**

Immune checkpoint inhibitor (ICI)-based regimens are transforming the landscape of hepatocellular carcinoma (HCC) treatment. We describe the effect of combined ipilimumab and nivolumab in patients with advanced HCC after the failure of prior ICI-based combination treatments.

**Methods:**

The clinical course of patients with advanced HCC who received combined ipilimumab and nivolumab after prior ICI-based combination therapies was assessed. Progression-free survival (PFS), overall response rate (ORR) and disease control rate (DCR) per RECIST v1.1 and mRECIST, overall survival (OS), and safety were analyzed.

**Results:**

Of 109 patients treated with atezolizumab and bevacizumab or other ICI-based combination treatments, ten patients received subsequent therapy with ipilimumab and nivolumab. The majority of patients had Barcelona Clinic Liver Cancer (BCLC) Stage C (80%) HCC and a preserved liver function as defined by Child–Pugh A (80%). At a median follow-up of 15.3 months, ORR for ipilimumab and nivolumab was 30% with a DCR of 40%. Median PFS was 2.9 months and the median OS was 7.4 months.

**Conclusion:**

This retrospective study demonstrates that combined ipilimumab and nivolumab can be effective and tolerable after prior ICI-based combination therapies and provides a rationale for the prospective clinical evaluation of this treatment sequencing.

**Supplementary Information:**

The online version contains supplementary material available at 10.1007/s00432-022-04206-8.

## Introduction

Hepatocellular carcinoma (HCC) is the sixth most common cancer worldwide with an increasing incidence and one of the world’s leading causes of cancer-related death (McGlynn et al. 2015; Torre et al. 2016; De Toni et al. 2020). Despite well-defined risk factors and established surveillance programs in patients with chronic liver disease (Kanwal and Singal 2019), most patients with HCC are diagnosed with tumors at advanced stages, where curative options are not feasible (Llovet et al. 2021). Sorafenib, a tyrosine kinase inhibitor (TKI), was the first agent to show significant clinical benefit in patients with advanced HCC (Llovet et al. 2008; Cheng et al. 2009). Since its approval in 2008, sorafenib has retained a central position in HCC treatment algorithms (Reig et al. 2021). Subsequent trials leading to the approval of new agents incorporated sorafenib either as a direct comparator or as a previous treatment line. Lenvatinib, another TKI, was approved in 2018 as an alternative treatment option for treatment-naïve patients with advanced HCC, based on the non-inferiority on overall survival as compared to sorafenib (Kudo et al. 2018). In addition, the two TKIs regorafenib, cabozantinib, and the vascular endothelial growth factor (VEGF) receptor 2 inhibitor ramucirumab became approved in the second-line after sorafenib based on the results of the respective pivotal trials (Zhu et al. 2015, 2019; Bruix et al. 2017; Abou-Alfa et al. 2018).

Checkpoint inhibitor-based immunotherapy marked a breakthrough in the treatment of a wide range of cancer entities, including HCC (Ribas and Wolchok 2018). Currently approved immune checkpoint inhibitors (ICI) are monoclonal antibodies enhancing the antitumor immune response by blocking the signaling mediated by programmed cell death 1 (PD-1) and its ligand PD-L1, or cytotoxic T lymphocyte antigen 4 (CTLA-4) (Leach et al. 1996; Iwai et al. 2005). The combination of the PD-L1 inhibitor atezolizumab and VEGF inhibitor bevacizumab markedly improved overall survival (OS) and progression-free survival (PFS) in patients with unresectable HCC as compared to sorafenib (Finn et al. 2020; Cheng et al. 2021). The combination became the first immunotherapeutic regimen for first-line therapy of patients with unresectable HCC, currently approved in more than 80 countries based on results of the IMbrave150 trial.

The approval of atezolizumab and bevacizumab has drastically changed the treatment paradigm of advanced HCC leading to a substantial improvement in patients’ survival, especially in patients with objective response to treatment (Finn et al. 2021). Nevertheless, the prognosis of patients with advanced disease remains dismal and there is an urgent need to establish evidence-based treatment options upon progression on atezolizumab and bevacizumab. Cabozantinib, regorafenib, and ramucirumab have been established within a schema of sequential treatment in patients who had received sorafenib as first-line treatment (Zhu et al. 2015; Bruix et al. 2017; Abou-Alfa et al. 2018). There are currently no data from large prospective trials on the efficacy of systemic therapies in patients with advanced HCC pretreated with atezolizumab and bevacizumab. Recent evidence from a retrospective study showed antitumor efficacy for TKIs after atezolizumab and bevacizumab (Yoo et al. 2021). Due to the efficacy of ICI in first-line treatment, it is expected that an ICI-based treatment regimen will be effective in the yet-to establish second-line setting. This is exemplified by the CheckMate 040 study showing a meaningful antitumor activity of ipilimumab and nivolumab with a mOS of 22.8 months in patients with advanced HCC after prior sorafenib, a regimen recently approved by the FDA in 2020 (Yau et al. 2020). However, very little data is available on the effect of ICI-based treatments after progression on ICI-based first-line therapies.

Here we describe the efficacy and safety of combined immune checkpoint blockade with ipilimumab and nivolumab in patients previously treated with atezolizumab plus bevacizumab or other ICI-based combinations.

## Materials and methods

### Patient population

We performed a retrospective study of patients with advanced HCC treated with ipilimumab and nivolumab after prior treatment with atezolizumab and bevacizumab or lenvatinib and nivolumab at three tertiary centers in Germany between 2020 and 2022. Patients had confirmed HCC diagnosis based on histopathological findings or typical diagnostic imaging as per European Association for the study of the Liver (EASL) criteria (European Association for the Study of the Liver 2018), with Barcelona Clinic Liver Cancer (BCLC) stage B, not amenable to curative treatment or locoregional therapy, or BCLC C (Cillo et al. 2006). Clinical, radiological, and laboratory data were extracted from electronic case records into a prospectively maintained database. Child–Pugh Score and Albumin–Bilirubin (ALBI) grade were used to analyze liver function, Eastern Cooperative Oncology Group (ECOG) scale to assess patients’ performance status. This study was approved by the ethics committee of LMU Munich and performed in accordance with the Declaration of Helsinki.

### Treatment schedule

All patients had prior treatment with combined atezolizumab and bevacizumab or nivolumab and lenvatinib. Atezolizumab was administered at a dose of 1200 mg and bevacizumab at 15 mg per kg of body weight intravenously (IV) every 3 weeks (Finn et al. 2020). Nivolumab was given IV at a dose of 240 mg every 2 weeks, lenvatinib at 12 mg orally once daily for patients with body weight ≥ 60 kg, and at 8 mg once daily for patients < 60 kg. Patients received ipilimumab and nivolumab after the failure of prior anti-PD-1/PD-L1 inhibitor-based combinations due to contraindications to multi-kinase inhibitors or due to progression after prior multi-kinase inhibitor therapy. Ipilimumab was administered at a dose of 3 mg and nivolumab at 1 mg per kg of body weight IV every three weeks for four doses (induction phase), followed by nivolumab 240 mg every 2 weeks (maintenance phase), in analogy to arm A of the Checkmate 040 trial (Yau et al. 2020). All patients received at least one dose of ipilimumab and nivolumab. Patients received no other concomitant antitumor treatment. Treatment decisions were made based on clinical, laboratory, and radiological assessments based on the recommendations of a multidisciplinary tumor board.

### Assessments

Patients received routine monitoring during immunotherapy with radiological assessments via computed tomography (CT) of the chest, abdomen, and pelvis or magnetic resonance imaging of the abdomen with CT chest every 12 weeks and alpha-fetoprotein (AFP) measurements. Tumor response was evaluated according to the Response Evaluation Criteria in Solid Tumors (RECIST) criteria version 1.1 (Eisenhauer et al. 2009) and modified RECIST for hepatocellular cancer (mRECIST) (Lencioni and Llovet 2010; Lencioni 2013) retrospectively by two experienced hepatobiliary radiologists.

### Endpoints

Progression-free survival (PFS) was defined as the time from treatment initiation to first disease progression on radiological assessment according to RECIST v1.1 or death from any cause. Overall response rate (ORR) consisted of the proportion of patients with RECIST v1.1-defined complete response (CR) or partial response (PR), disease control rate in the proportion of patients with CR, PR, or stable disease (SD). Progressive disease (PD) was defined as radiological progression per RECIST v1.1 criteria or cancer-related clinical deterioration. Patients were followed up after the last administration of ipilimumab and nivolumab. Safety evaluations included assessments of type, incidence, and severity of adverse events (AE) according to National Cancer Institute Common Terminology Criteria for Adverse Events (NCI CTCAE) version 4.

### Statistics

All analyses were performed using GraphPad Prism 8 Software (GraphPad Software, San Diego, CA, USA). Baseline characteristics and response rates were summarized in a descriptive manner. Median and range were used for numerical variables and frequencies and percentages for categorical variables. The cut-off date for follow-up was May 1, 2022, and median follow-up time was calculated using Reverse Kaplan–Meier analysis. Median PFS and OS were estimated by Kaplan–Meier analysis.

## Results

### Patient population

Between January 2020 and January 2022, 109 patients with advanced HCC were treated with atezolizumab and bevacizumab or nivolumab and lenvatinib. Of those, a total of ten patients received subsequent therapy with ipilimumab and nivolumab while 36 patients were still on their initial immunotherapeutic regimen (see online resource 2). 73 patients discontinued the first PD-1/PD-L1 inhibitor-based combinations but did not go on to receive ipilimumab + nivolumab. Reasons included availability of other approved alternatives without contraindications, clinical deterioration without any further oncological treatment, death or loss to follow-up. Baseline characteristics are listed in Table 1. The median age was 63 (range 31–82) and 60% of patients (*n* = 6) were female. The majority of patients had advanced HCC BCLC C (80%, *n* = 8) due to extrahepatic spread (EHS) (70%, *n* = 7) and/or macrovascular invasion (MVI) (30%, *n* = 3) with preserved liver function defined by Child–Pugh A (80%, *n* = 8) with ALBI grade 1 (70%, *n* = 7); and suffered from underlying cirrhosis (60%, n = 6) due to chronic viral hepatitis (50%, *n* = 3), or non-viral etiologies (50%, *n* = 3). Patients with autoimmune diseases or organ transplant recipients were not present in the study population. ECOG of 0–1 was present in 90% of patients (*n* = 9) and AFP ≥ 1000 μg/L in 60% (*n* = 6). Most patients received prior therapy for HCC, including surgery, locoregional therapy, and multi-kinase inhibitors.

**Table 1:** Patient baseline characteristics

Median age, range (years)	62.5 (31–81)
Female, *n* (%)	6 (60%)
HCC etiology, *n* (%)	
Hepatitis B	2 (20%)
Hepatitis C	1 (10%)
Alcoholic	1 (10%)
NASH	1 (10%)
Idiopathic/non-cirrhotic	4 (40%)
Other	1 (10%)
BCLC stage, *n* (%)	
*B*	1 (10%)
*C*	8 (80%)
*D*	1 (10%)
Extrahepatic metastases, *n* (%)	7 (70%)
Macrovascular invasion, *n* (%)	3 (30%)
AFP ≥ 1000 μg/L, *n* (%)	6 (60%)
Child–Pugh grade	
*A*	8 (80%)
*B*	1 (10%)
*C*	1 (10%)
ALBI grade	
1	7 (70%)
2	2 (20%)
3	1 (10%)
Baseline ECOG performance status	
0–1	9 (90%)
2	1 (10%)
Prior IO combination therapy	
Atezolizumab with bevacizumab	7 (70%)
Nivolumab with lenvatinib	3 (30%)
Best response to prior IO combination therapy by RECIST v1.1	
SD	6 (60%)
PD	4 (40%)
Lines of systemic therapies prior to ipilimumab + nivolumab, *n* (%)	
1	5 (50%)
2	1 (10%)
3 or more	4 (40%)
Therapies prior to any IO, *n* (%)	
Prior local ablation	1 (10%)
Prior surgery	4 (40%)
Radiotherapy/TARE	2 (10%)
TACE	1 (10%)
Prior systemic treatment with TKI	5 (50%)
Sorafenib	5 (50%)
Cabozantinib	4 (40%)
Regorafenib	1 (10%)

*ALBI* albumin–bilirubin grade, *AFP* alpha-fetoprotein, *BCLC* Barcelona Clinic Liver Cancer, *ECOG* Eastern Cooperative Oncology Group, *HCC* hepatocellular carcinoma, *IO* immunotherapy, *NASH* non-alcoholic steatohepatitis, *PD* progressive disease, *RECIST v1.1* Response Evaluation Criteria in Solid Tumors version 1.1, *SD* stable disease, *TACE* transarterial chemoembolization, *TARE* transarterial radioembolization, *TKI* tyrosine kinase inhibitor

### Characteristics of prior ICI-based combination treatment

All patients received either prior combination therapy with atezolizumab and bevacizumab (70%, *n* = 7) or nivolumab and lenvatinib (30%, *n* = 3). Nine patients (90%) experienced disease progression upon prior ICI-based combinations, while in one patient, therapy had to be discontinued due to severe toxicity (hypertension grade IV). Median progression-free survival in these patients was 5.1 months (range 2.5–8.7) (online resource 1). Of note, none of the patients showed a radiological response to prior immunotherapy. Immune-related adverse events occurred in one patient (10%, *n* = 1) treated with atezolizumab and bevacizumab, consisting of exanthema CTCAE grade II. The median interval between the last dose of the prior ICI-based combination and ipilimumab plus nivolumab was 1 month (range 1–13).

### Outcomes

**Fig. 1:**
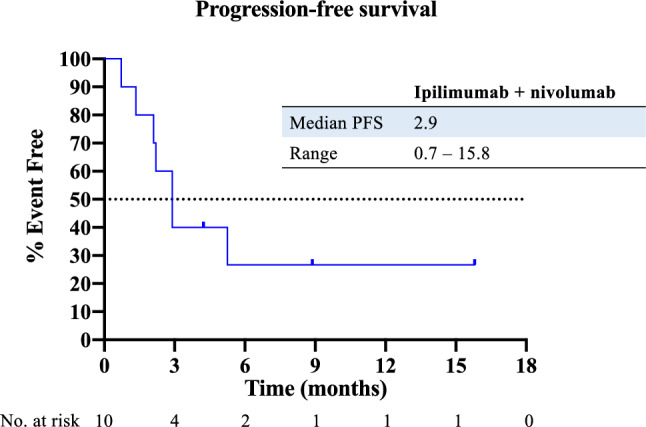
Kaplan–Meier analysis of progression-free survival of patients with advanced HCC treated with ipilimumab and nivolumab after the failure of prior PD-1/PD-L1 inhibitor-based combination therapy. *PFS* progression-free survival

**Fig. 2:**
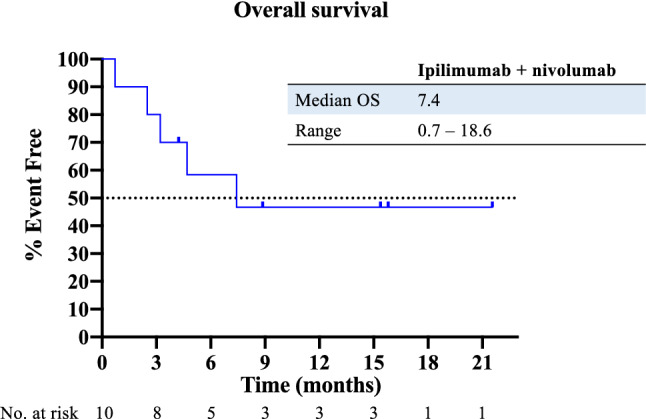
Kaplan–Meier analysis of overall survival of patients with advanced HCC treated with ipilimumab and nivolumab after the failure of prior PD-1/PD-L1 inhibitor-based combination therapy. *OS* overall survival

**Fig. 3:**
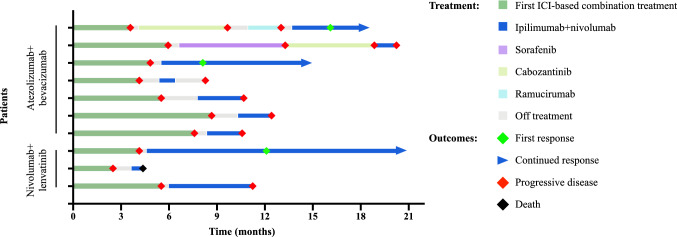
Swimmers blot illustrating the clinical course of 10 patients with advanced HCC treated with sequential combination immunotherapy. The blot depicts the time from initiation of PD-1/PD-L1 inhibitor-based combination therapy followed by ipilimumab and nivolumab until progression or death. The name of the combination therapy prior to ipilimumab and nivolumab (atezolizumab + bevacizumab or nivolumab + lenvatinib) is indicated at the beginning of the y-axis

Patients were observed for a median of 15.3 months at data cut-off. Ipilimumab and nivolumab was administered in five patients (50%) as second-line therapy, while the other five patients received it in later lines (Table 1). Median progression-free survival was 2.9 months (Fig. 1), whereas median overall survival was 7.4 months (Fig. 2). Response to treatment as per RECIST v1.1 and mRECIST is depicted in Table 2. ORR was 30% per RECIST v1.1 and mRECIST, with one patient achieving complete response and two patients having a partial response (PR) per mRECIST, while stable disease occurred in one patient (10%) and progressive disease in six patients (60%), respectively. Disease control was achieved in four patients (40%). The patients with CR/PR continued responding at data cut-off (Fig. 3). To investigate a possible influence of response to prior PD-1/PD-L1 inhibitor-based combination treatments on the efficacy of ipilimumab and nivolumab, patients were divided into two groups: (1) patients with durable disease control from prior PD-1/PD-L1 inhibitor-based combination, defined as time of disease control ≥ 5.1 months (*n* = 5), and (2) patients refractory to prior PD-1/PD-L1 inhibitor-based combination, defined as time of disease control < 5.1 months (*n* = 5). Interestingly, the three responders to ipilimumab and nivolumab belonged to the group of patients who showed a shorter disease control on prior PD-1/PD-L1 inhibitor-based treatment. All responders had non-viral HCC BCLC C with MVI/EHS, a preserved liver function with Child–Pugh A and a good performance status of ECOG 0–1. 2 of 3 had received surgery, locoregional treatment and sorafenib prior to any immunotherapies.

**Table 2:** Overall response per RECIST v1.1 and mRECIST

Variable	RECIST v1.1 % (*n*)	mRECIST % (*n*)
Overall response rate	30 (3)	30 (3)
Complete response	0 (0)	10 (1)
Partial response	30 (3)	25 (2)
Stable disease	10 (1)	10 (1)
Disease control rate	40 (4)	40 (4)
Progressive disease	60 (6)	60 (6)
Ongoing response at cut-off	30 (3)	30 (3)

*mRECIST* modified Response Evaluation Criteria in Solid Tumors, *RECIST v1.1* RECIST version 1.1

### Safety data

The median number of administered cycles of ipilimumab and nivolumab was four (range 1–30). Six patients (60%) completed the induction phase with ipilimumab and nivolumab. No dose adjustments were performed. In seven cases (70%) therapy was discontinued due to progressive disease (Fig. 3), with two patients experiencing rapid clinical deterioration due to cancer progression in the first two months after initiation of ipilimumab and nivolumab. Immune-related adverse events (irAEs) were observed in four patients (40%). Two patients experienced low-grade skin toxicity (rash, pruritus), two patients had diarrhea/colitis grade II, and another hypophysitis grade III. Treatment was interrupted in one patient with colitis grade II, which resolved after the administration of steroids. No patient had to discontinue immunotherapy due to irAEs. Three patients had non-immune-mediated adverse events leading to treatment interruptions, two due to fatigue grade III and one patient due to hypercalcemia grade III, respectively. Of note, all patients experiencing immune-mediated toxicity achieved clinical benefit from ipilimumab and nivolumab defined by CR, PR, or SD (mRECIST).

### Subsequent treatments

At the end of the follow-up, five patients were still alive (50%) and the three responders continued receiving ipilimumab and nivolumab (30%) (Fig. 3). Of the seven patients (70%) with disease progression upon ipilimumab and nivolumab, four (57%) received subsequent systemic therapy. Two patients received multi-kinase inhibitors (lenvatinib, sorafenib), and two patients a combination of lenvatinib and anti-PD-1 inhibitor pembrolizumab.

## Discussion

Treatment with atezolizumab and bevacizumab has greatly improved the prognosis of patients with advanced HCC (Finn et al. 2021). Most recently, the positive HIMALAYA trial has established the combination of durvalumab and tremelimumab as an additional ICI-based regimen for first-line treatment of advanced HCC (Abou-Alfa et al. 2022), and other PD-1/PD-L1 inhibitor-based combinations are being investigated (Llovet et al. 2019; Mueller et al. 2020). However, despite the improvements in patients’ survival, a high percentage of patients will not respond to treatment and finally progress requiring further systemic therapy (Finn et al. 2021). As several different regimens of ICI-based options of combined treatment have shown promising results in early-phase clinical studies, choosing the best regimen for second-line treatment after failure of the first-line treatment with ICI represents a key challenge for the design of clinical trials and in clinical practice. The efficacy of the anti-CTLA-4/anti-PD-1 combination ipilimumab and nivolumab after the failure of a prior PD-1/PD-L1 inhibitor-based treatment in patients with advanced HCC has not been reported yet.

This retrospective study shows that sequential therapy with ipilimumab and nivolumab can lead to meaningful and durable responses in anti-CTLA-4-naïve patients after failure of prior PD-1/PD-L1 inhibitor-based combinations. Dual immune checkpoint blockade with ipilimumab and nivolumab has been studied in cohort 4 of the CheckMate 040 trial (Yau et al. 2020). In this trial, patients with advanced HCC previously treated with sorafenib were randomized to receive ipilimumab and nivolumab in one of three different dosing schemes. The regimen of arm A consisting of nivolumab 1 mg/kg and ipilimumab 3 mg/kg showed the most marked benefit with a median OS of 22.8 months and was approved by the FDA in this indication. The response rates reported in the CheckMate 040 trial and our retrospective study are similar at 32 and 30%, respectively (Yau et al. 2020). It is well documented that responses to immune checkpoint inhibitors tend to be more durable than in other types of systemic therapies (Pons-Tostivint et al. 2019). In around 60% of the patients receiving ipilimumab and nivolumab in the CheckMate 040 trial, responses lasted longer than one year and the median duration of response (DOR) in arm A was not reached (Yau et al. 2020). The three responders showed a durable response at the data cut-off in our series. Median OS in our cohort was shorter as compared to the outcomes of the CheckMate 040 trial with 7.4 vs. 22.8 months, which is consistent with previous reports on real-world efficacy of immunotherapy in HCC and reflects the more heterogeneous real-life populations (Finkelmeier et al. 2019). Our cohort was substantively different from the CheckMate 040 trial population including patients with an impaired liver function, bad performance status, and heavily pretreated patients. Despite these factors, we still observed responses in 30% of the cases. Interestingly, all responders belonged to the patients experiencing a shorter duration of response upon prior PD-1/PD-L1 inhibitor-based combination. This finding could point to different immunological mechanisms behind the action of PD-1/PD-L1 inhibitor-based combination therapies and anti-CTLA-4 combinations in HCC. First, inhibition of CTLA-4 enhances early activation and proliferation of effector T cells upon tumor neoantigen recognition in the lymph nodes (Rooij et al. 2013; Snyder et al. 2014; Ribas and Wolchok 2018). Duffy et al. demonstrated that intratumoral CD8+ T cells accumulate in HCC patients upon CTLA4-inhibition (Duffy et al. 2017). Second, CTLA-4 blockade depletes immunosuppressive regulatory T cells in the tumor microenvironment (Sharma et al. 2019). By these mechanisms, ipilimumab and nivolumab might rescue resistance to prior PD-1/PD-L1 inhibitor-based combination treatment and switch an immunotherapy-resistant to a immunotherapy-sensitive phenotype. The immunological phenomena behind the observed efficacy of ipilimumab and nivolumab upon prior PD-1/PD-L1 inhibitor-based combinations require further studies.

Our data indicate that ipilimumab and nivolumab could be effective not only after prior therapy with sorafenib but also after prior PD-1/PD-L1 inhibitor-based combinations such as atezolizumab and bevacizumab. Data on combined checkpoint blockade in HCC following prior immunotherapy are scarce. A recent study reported outcomes of 25 patients with advanced HCC treated with ipilimumab and PD-1 inhibition with pembrolizumab or nivolumab after prior anti-PD-1/PD-L1 monotherapy (Wong et al. 2021). The authors reported a considerably lower ORR of 16% as compared to the CheckMate 040 trial or our data, while responses were durable with a DOR of 11.5 months. Apart from different pre-treatments, there are differences between this study and our analysis. First, patients received either ipilimumab at a low dose of 1 mg/kg and pembrolizumab at 2 mg/kg or nivolumab at 3 mg/kg. This deviates from the FDA approval of ipilimumab 3 mg/kg and nivolumab 1 mg/kg (Food and Drug Administration Washington D.C. U.S. 2022). The combination of ipilimumab and pembrolizumab has not been studied in prospective trials, to our knowledge. Second, the number of HBV-infected patients was markedly higher in the study by Wong et al. as compared to the CheckMate 040 trial or our cohort. However, since both studies suggest antitumor activity of CTLA-4/PD-1 inhibition after prior immunotherapy, prospective studies are urgently needed to elucidate this question. Of note, no trial is currently investigating this treatment sequence.

Dual immune checkpoint inhibition targeting anti-PD-1/PD-L1 and anti-CTLA-4 showed a higher efficacy as compared to monotherapy in the second-line therapy of HCC (Yau et al. 2020; Kelley et al. 2021). However, the increased antitumor activity is accompanied by a higher rate of immune-mediated toxicity (Yau et al. 2020; Kelley et al. 2021). Encouragingly, the rate of irAE in our cohort was low, with only one patient experiencing grade 3 hypophysitis and no treatment-related death or the need for treatment discontinuation due to irAEs. However, the lower rate of adverse events might be due to the retrospective nature of our study. A possible association between irAEs and response has been reported for immunotherapies across many malignancies, including HCC (Das and Johnson 2019). Interestingly, all patients benefitting from the anti-PD-1/anti-CTLA-4 combination in this study also experienced immune-mediated toxicity. Notably, one patient with impaired liver function did not benefit dying shortly after treatment initiation. This underlines that the indication to use nivolumab and ipilimumab in HCC with decompensated liver function should be strict, even if a recent trial suggests, that monotherapy with nivolumab might be safe in patients with child–Pugh B liver function (Kudo et al. 2021).

The most obvious limitation of our study is the small number of patients our results are based. This is because combined ipilimumab and nivolumab are currently not approved in Europe for second-line therapy of advanced HCC (European Medicines Agency 2022). The use of this combination is thus restricted to patients presenting with contraindications to standard second-line treatments on a single-case basis. The second limitation is the retrospective nature of this study, which could lead to underreporting of safety data. Nevertheless, our study provides to our knowledge the first data on the use of ipilimumab and nivolumab in patients who had previously received ICI-based combination regimens.

## Conclusions

In summary, our study shows that ipilimumab and nivolumab can have meaningful antitumoral activity in a relevant proportion of patients with advanced HCC after the failure of prior PD-1/PD-L1 inhibitor-based combinations like atezolizumab and bevacizumab. Real-world accounts like ours may help design prospective, randomized clinical trials of second-line treatment. This report may also provide guidance for the choice of second-line treatments after prior immunotherapy on a single-case basis when the currently established treatment options are not feasible or not available.

## Supplementary Information

Below is the link to the electronic supplementary material.

Supplementary file1 (EPS 947 KB)

Supplementary file2 (EPS 1065 KB)

## Data Availability

All data being analyzed as part of this study are included in this manuscript and the supplementary materials. Further inquiries can be sent to the corresponding authors.
